# Synthesis, Structure and Impact of 5-Aminoorotic Acid and Its Complexes with Lanthanum(III) and Gallium(III) on the Activity of Xanthine Oxidase

**DOI:** 10.3390/molecules26154503

**Published:** 2021-07-26

**Authors:** Lozan Todorov, Luciano Saso, Khedidja Benarous, Maria Traykova, Abderahmane Linani, Irena Kostova

**Affiliations:** 1 Department of Chemistry, Faculty of Pharmacy, Medical University, 1000 Sofia, Bulgaria; irenakostova@yahoo.com; 2 Department of Physiology and Pharmacology “Vittorio Erspamer”, Faculty of Pharmacy and Medicine, Sapienza University, 00185 Rome, Italy; luciano.saso@uniroma1.it; 3 Laboratoire des Sciences Fondamentales, Université Amar Telidji, Laghouat 03000, Algeria; k.benarous@lagh-univ.dz (K.B.); abde.linani@lagh-univ.dz (A.L.); 4 Department of Physics and Biophysics, Faculty of Medicine, Medical University, 1431 Sofia, Bulgaria; mlvtraykova@gmail.com

**Keywords:** lanthanum, gallium, coordination complex, superoxide, xanthine oxidase, enzyme inhibition, molecular docking

## Abstract

The superoxide radical ion is involved in numerous physiological processes, associated with both health and pathology. Its participation in cancer onset and progression is well documented. Lanthanum(III) and gallium(III) are cations that are known to possess anticancer properties. Their coordination complexes are being investigated by the scientific community in the search for novel oncological disease remedies. Their complexes with 5-aminoorotic acid suppress superoxide, derived enzymatically from xanthine/xanthine oxidase (X/XO). It seems that they, to differing extents, impact the enzyme, or the substrate, or both. The present study closely examines their chemical structure by way of modern methods—IR, Raman, and ^1^H NMR spectroscopy. Their superoxide-scavenging behavior in the presence of a non-enzymatic source (potassium superoxide) is compared to that in the presence of an enzymatic source (X/XO). Enzymatic activity of XO, defined in terms of the production of uric acid, seems to be impacted by both complexes and the pure ligand in a concentration-dependent manner. In order to better relate the compounds’ chemical characteristics to XO inhibition, they were docked in silico to XO. A molecular docking assay provided further proof that 5-aminoorotic acid and its complexes with lanthanum(III) and gallium(III) very probably suppress superoxide production via XO inhibition.

## 1. Introduction

The importance of the superoxide radical, O_2_^−^, for human health and disease has been known for a long time [1,2,3]. It plays a crucial role in normal biological processes [4,5,6,7,8] as well as in the initiation of pathological conditions [9,10,11,12,13,14,15,16]. Extracellular superoxide radicals are characteristically released by cell types involved in immune defense, and also by many additional types of cells [17,18,19]. The formation of O_2_^−^ is inevitable in the living body. It is involved in a number of normal physiological processes as well as in cellular malignization, tumor proliferation, and malignant cell death [20,21].

The involvement of superoxide in cancer onset [22,23,24,25], development [26], and progression [27] is complex [28] and intensively explored [29]. Oxidative stress (OS)—the metabolic accumulation of free radicals dominating due to their deficient elimination—is typical for cancer cells [21,30]. OS is involved in carcinogenesis. It is also capable of helping eradicate malignant cells by varying their redox homeostasis.

Xanthine oxidase (XO) is one of the superoxide-producing enzymes. It is a homodimer with a molecular mass of 290 kDa. Belonging to the molybdenum protein family, it consists of two independent subunits, containing two separated substrate-binding sites [31]. Each subunit contains one flavin adenine dinucleotide (FAD), one molybdenum, and two iron–sulfur (2Fe-2S) centers of the ferredoxin type. XO catalyzes the oxidation of hypoxanthine to xanthine and subsequently to uric acid [32,33,34]. Throughout the reoxidation of XO, molecular oxygen acts as an electron acceptor, producing superoxide and hydrogen peroxide [35]. Control over superoxide formation and accumulation in the human body is essential for human health, therefore any effect on the activity of XO may impact the maintenance of healthy homeostasis.

Different approaches to control XO-generated superoxide have been explored in the search for efficient malignant cell elimination [36,37,38]. The goal of chemotherapy is to kill tumors by initiating apoptotic death [39,40,41]. That is achieved by raising oxidative stress levels in the tumor using a variety of drugs with pro-oxidant properties [42].

Lanthanide (Ln) ions induce cell membrane perforation and apoptosis, increase ROS-mediated oxidative damage, and alter the assembly and stability of the cytoskeleton. These effects make them potential pharmacological agents in cancer therapy [43]. Successful transport of lanthanide ions to the tumor has to be accompanied by some kind of mechanism to avoid their detrimental effect on healthy cells. These somewhat contradicting requirements characterize the search for new coordination compounds with improved antitumor activity and protective effects on normal cells [44,45]. One possible solution would be to use a coordination complex, comprising a highly pro-oxidant lanthanide cation, surrounded by bioactive ligands with antioxidant capacity. Such a complex has to be stable at a neutral, homeostatic pH and, at the same time, has to dissociate at acidic pH values that are typical for tumors. A number of complexes of gallium (Ga) and lanthanum (La) have been investigated in recent years for their pharmacological activities [46,47,48,49,50]. The Ga(III) and La(III) complexes with 5-aminoorotic acid have been shown to be stronger in vitro antioxidants, compared to the free ligand, at a normal, homeostatic pH [51,52,53,54].

In our previous investigations, we observed differing in vitro interactions of 5-aminoorotic acid and its complexes with superoxide, generated by different model systems [54,55]. Possible interactions of the compounds investigated with components of the superoxide-generating model systems were proposed. The aim of the present investigation is to look for a possible reason for the disparate superoxide-scavenging activity of 5-aminoorotic acid and its complexes with Ga(III) and La(III) in the presence of one non-enzymatic potassium superoxide (KO_2_) and one enzymatic xanthine/xanthine oxidase model systems. In both model systems, superoxide was the only free radical generated and its elimination was monitored using luminol-dependent chemiluminescence (LDCL). The radical-scavenging activity of a compound in the presence of the X/XO model system was compared with the activity of uric acid (UA) formation of the enzyme in the presence of the compound, estimated using UV spectroscopy.

A computer model of the interaction of 5-aminoorotic acid (HAOA) and its complexes with Ga(III)—GaAOA—and La(III)—LaAOA—with xanthine oxidase was developed using ChemDraw 16.0—a tool for generating chemical and biological drawings. It is the preferred tool for drawing chemical and biological concepts with metal atoms based on IUPAC, IUBMB, and CAS rules. It allows for calculating more than 100 chemical properties of a given structure. The complexes used in the work were prepared based on the Lewis and the Cahn–Ingold–Prelog (CIP) rules for stereochemistry structure and all the complexes were prepared and checked automatically by ChemDraw and saved in mol.2 format.

The complexes of lanthanum(III) and gallium(III) were synthesized by a reaction of Ga(III) and La(III) nitrates with 5-aminoorotic acid in amounts equal to a metal:ligand molar ratio of 1:3. The structures of the synthesized complexes were determined by means of spectral (FT-IR, FT-Raman, ^1^H NMR) and elemental analysis. Since the crystal structure data are not available, theoretical approaches for determination of the geometrical parameters, vibrational frequencies, and the binding mode for the model M(III)–AOA at a high level of theory are very helpful for extracting reliable structural information. Significant differences in the bands of the IR spectra of the complexes were observed and compared to the spectrum of the free ligand. A comparative analysis of the Raman spectra of the metal complexes with that of 5-aminoorotic acid allowed a good assignment of the vibrations of the functional groups involved in the coordination. The vibrational analysis, performed for the investigated species—5-aminoorotic acid and its metal complexes—helped explain the vibrational modes of the ligand, sensitive to interaction with the metals. The detailed vibrational study provided indication of the coordination behavior of the ligand to metal ions being in agreement with literature data and theoretical predictions.

## 2. Results

### 2.1. Chemistry

The compositions of the newly obtained metal complexes were characterized by elemental analysis. The nature of Ga(III) and La(III) complexes was confirmed by IR, Raman, and ^1^H NMR spectroscopy. The data from the elemental analysis of the obtained compounds served as a basis for the determination of their empirical formulas: M(AOA)_3_·3H_2_O, where M = La, Ga and AOA^−^ = C_5_N_3_O_4_H_4_^−^.

The elemental analysis data of La(AOA)_3_·3H_2_O are shown as % calculated/found: C = 25.60/25.14; H = 2.56/2.24; N = 17.92/17.48; H_2_O = 7.68/7.25; La: 19.77/20.06, and for Ga(AOA)_3_·3H_2_O: C = 28.39/28.56; H = 1.89/2.02; N = 19.87/20.05; H_2_O = 8.52/8.34; Ga = 10.99/11.23, where HAOA = C_5_N_3_O_4_H_5_ and AOA = C_5_N_3_O_4_H_4_^−^.

#### 2.1.1. Vibrational Spectroscopy

**Figure 1:**
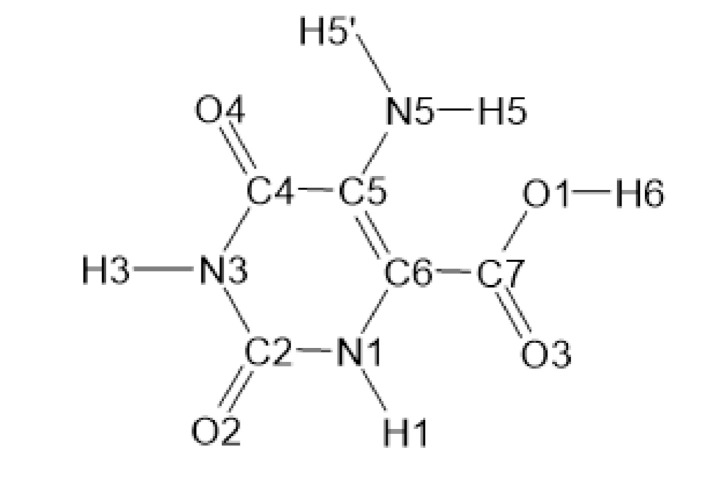
Chemical structure of 5-aminoorotic acid.

The vibrational band assignments obtained from the IR and Raman spectra were analyzed by comparing them with those from the literature [56,57,58,59,60,61,62,63,64,65,66,67,68,69,70,71,72,73,74,75,76,77,78,79] and they were in agreement with the results of our DFT calculations (i.e., harmonic vibrational wavenumbers and Raman scattering activities) [80]. In Table 1, the selected calculated and experimental IR and Raman data in conjunction with their tentative assignment are given. The chemical structure of 5-aminoorotic acid is presented in Figure 1.

In the spectral region from 3600–2000 cm^−1^ of the IR spectrum, the C-H and N-H stretches give rise to medium IR bands. In the IR spectra, the strong band at 3457 cm^−1^ (for the 5-aminoorotic acid) and the medium bands for the La(III) and Ga(III) complexes were assigned to the N-H stretching modes of the pyrimidine rings (Table 1), while the band at 3333 cm^−1^ (strong) in the IR spectrum of 5-aminoorotic acid, along with the band at 3348 cm^−1^ (weak/medium) of the IR spectrum of the La(III) complex, and the band at 3336 cm^−1^ (medium) of the IR spectrum of the Ga(III) complex, were attributed to the C-H stretching modes [56,74] (Table 1). In the Raman spectra of the ligand and metal complexes, the C-H and N-H vibrations were observed as follows: the N3-H3 stretching mode at 3456 cm^−1^ and the C-H stretching mode at 3323 cm^−1^ (Raman spectrum of the ligand); the N1-H1 stretching mode at 3478 cm^−1^ (Raman spectrum of the La(III) complex) and the C-H stretching mode at 3334 cm^−1^ (Raman spectrum of the Ga(III) complex). The asymmetrical NH_2_ stretch, absent in the IR spectrum of the ligand, can be seen in the vibrational spectra of the complexes at 3361 cm^−1^ and 3355 cm^−1^ as medium bands (IR spectra of La(III) and Ga(III) complexes) and at 3357 cm^−1^ and 3358 cm^−1^ as weak bands (Raman spectra of La(III) and Ga(III) complexes), whereas the symmetrical NH_2_ stretch can be detected in the IR spectra of the ligand and metal complexes by signals with medium intensities, at 3196 cm^−1^ (ligand), 3171 cm^−1^ (La(III) complex), 3168 cm^−1^ (Ga(III) complex) and only in the Raman spectrum of the ligand as a very weak band at 3166 cm^−1^ [60]. The 2700–3000 cm^−1^ wavenumber region of the IR spectra of 5-aminoorotic acid and its complexes is characteristic of strongly hydrogen-bonded intermolecular complexes [60,61,62].

A very strong band at 1691 cm^−1^ in the IR spectrum of 5-aminoorotic acid and two strong bands at 1718 cm^−1^ and 1684 cm^−1^ in the IR spectrum of the La(III) complex and at 1717 cm^−1^ and 1691 cm^−1^ in the IR spectrum of the Ga(III) complex could be detected in the 1730-1690 cm^−1^ region, which were assigned to the symmetrical C2=O2 and to the N-H stretching modes. Opposite to the IR spectra, in the same region of the Raman spectra, only one medium band at 1698 cm^−1^ for the ligand was observed.

The detected strong band at 1667 cm^−1^ (IR spectrum of 5-aminoorotic acid) and the two very strong bands for each of the complexes, at 1673 cm^−1^ and 1637 cm^−1^ (IR spectrum of La(III) complex) and at 1676 cm^−1^ and 1645 cm^−1^ (IR spectrum of Ga(III) complex), were assigned to the symmetrical stretching C4=O4 modes [62] and to the asymmetrical COO^-^ stretching modes [60,68]. In the respective Raman spectra, these vibrations can be found as a shoulder at 1678 cm^−1^ for the ligand, as a medium band at 1673 cm^−1^ for its La(III) complex, and as a medium band at 1682 cm^−1^ for its Ga(III) complex (Table 1). In the IR spectrum of 5-aminoorotic acid, the strong band at 1604 cm^−1^ was attributed to the C5-C6 stretching contributions and NH_2_ scissoring [60,62], whereas in the IR spectra of the metal complexes are absent. In the Raman spectra, these vibrations are presented as very strong bands at 1612 cm^−1^ for 5-aminoorotic acid and at 1623 cm^−1^ for its La(III) complexes and 1628 cm^−1^ for its Ga(III) complexes (Table 1).

The IR bands with medium relative intensities at 1566 cm^−1^ (ligand), 1556 cm^−1^, and 1553 cm^−1^ (La(III) and Ga(III) complexes), as well as the medium bands in the Raman spectra, at 1560 cm^−1^ (ligand), 1542 cm^−1^, and 1546 cm^−1^ (La(III) and Ga(III) complexes), were assigned to the C5-C6 stretching and in plane N-H bending modes (Table 1). The pyrimidine ring vibrations (N-C and N-H bending modes) were found as follows: a weak peak at 1511 cm^−1^ (IR spectrum of 5-aminoorotic acid), a medium band at 1499 cm^−1^ (IR spectrum of the La(III) complex), and a medium signal at 1498 cm^−1^ (IR spectrum of the Ga(III) complex), while in the Raman spectra these vibrational modes were detected for the ligand as a weak/medium band at 1492 cm^−1^ and as weak signals at 1494 cm^−1^ and 1501 cm^−1^ for its La(III) and Ga(III) complexes, respectively (Table 1). Other in-plane N-H bending modes were observed only in the IR and Raman spectra of the ligand or in both spectra types (Table 1). The symmetrical COO^-^ stretching mode was detected in the IR spectra as a medium signal at 1405 cm^−1^ for 5-aminoorotic acid and as strong bands at 1390 cm^−1^ and 1391 cm^−1^ for its La(III) and Ga(III) complexes, while in the Raman spectra this vibrational mode appears only for the metal complexes as strong signals at 1384 cm^−1^ and 1388 cm^−1^ (La(III) and Ga(III) complexes, Table 1).

The very weak band at 989 cm^−1^ in the ligand IR spectrum (absent in the IR spectra of the complexes), which corresponds to the trigonal pyrimidine ring breathing mode [56,60], cannot be observed in the respective Raman spectra of the studied compounds. The weak band in the ligand IR spectrum (924 cm^−1^) and the very weak bands at 941 and 944 cm^−1^ in the IR spectra of the metal complexes, as well as the weak/medium band at 919 cm^−1^ (Raman spectrum of the ligand) and the weak bands at 933 and 948 cm^−1^ in the metal complex Raman spectra, were assigned to the out of plane C-C and C-N bending modes.

As a whole, from the comparison between the vibrational spectra of the ligand and the comparable spectra of the metal complexes, we can emphasize wavenumber shifting, with increases and/or decreases in their relative intensities, as well as appearances and/or disappearances of several bands. The observed changes can be caused by the decrease in the force constants of the C-C and C-H bonds and their polarization in the pyrimidine rings [71]. The complex formation with metals perturbs the ring aromatic and quasi-aromatic systems [81]. The metal affects the metal–oxygen bonds, and this effect is relocated to the C-O bonds. Subsequently, the force constant of the OCC_ring_ bond is changed, which reproduces the displacement of the electronic charge around bonds between heterocyclic rings, and heterocyclic rings and protons [82].

The ligand’s pyrimidine ring bending vibrations and the skeletal deformation bands in the 900–300 cm^−1^ wavenumber region show significant changes in coordination (Table 1). These changes may be connected with the distortion of the pyrimidine rings upon complex formation. Additionally, the spectra in the frequency region under 600 cm^−1^ are quite interesting, since they offer information about metal–ligand vibrations. The new detected bands in the complexes’ IR spectra, at 617 cm^−1^ (La(III) complex) and at 602 cm^−1^ (Ga(III) complex), as well as the new shoulders at 521 cm^−1^ (La(III) complex) and 509 cm^−1^ (Ga(III) complex), which are not observable in the Raman spectra, can be attributable to the M-O interactions [60,62,79]. The metal disturbs the carboxylate anion as well as the whole ring structure. The metal ionic potential is the most significant parameter, responsible for the metal influence on the rest of the organic molecule [82,83,84]. It is known that carboxylic acids interact with the metals as symmetric [85,86], bidentate carboxylate anions and both oxygen atoms of the carboxylate are symmetrically bonded to the metal [87]. In this sense, we could detect, in the low-wavenumber region of the Raman spectra of the La(III) and Ga(III) complexes, weak bands at 224, 192 cm^−1^ and 205, 182 cm^−1^, respectively, which can be attributed to the O1-M-O3 vibration modes (Table 1) [88,89,90].

#### 2.1.2. ^1^H NMR Spectra of the Ligand and Its Metal Complexes

The coordination of the metal ions with the ligand’s carboxylate oxygen atoms was demonstrated with the help of the data of ^1^H NMR spectra. Proton spectra recorded at 250 MHz in DMSO-*d_6_* confirmed complex formation. The typical chemical shifts of the ^1^H NMR spectra in DMSO-*d_6_* are presented in Table 2.

The ^1^H NMR spectrum of 5-aminoorotic acid in DMSO-*d_6_* demonstrates the expected three resonances which correspond to the NH protons. The carboxamido and imido protons have singlets at δ_H_ 11.47 and 9.44 and the amino protons provide a broad signal at δ_H_ 6.00 [72]. The amido and imido protons were also observed in the spectra of the complexes in the same solvent. The complexes’ spectra show a very broad peak at ca. δ_H_ 3.4, which can be attributed to the intermolecular exchange of protons between the amino group and water (contained in the solvent). We did not observe two sharp signals of the separated species or an averaged signal which could be due to the intermediate rate of the exchange [91,92]. The observed N_3_-H proton resonance in the NMR spectra (Table 2) clearly shows that this nitrogen atom is not involved in complex formation. Therefore, we can conclude that M(III) ions appear to bind the 5-aminoorotic acid at the carboxyl ion, as reported for most of the studied orotato complexes [68,71,91,92].

It can be concluded that, as has been reported previously for analogous complexes [72], the ^1^H NMR spectra of the new La(III) and Ga(III) complexes in DMSO-*d_6_* showed the presence of one carboxamido, one imido, and two amino protons. Additionally, the complexes’ signals in DMSO-*d_6_* are quite similar in the regions of the NH and NH_2_ proton resonances. Proton NMR spectra of 5-aminoorotic acid and its La(III) and Ga(III) complexes confirmed the anticipated coordination of 5-aminoorotic acid via its carboxylate oxygen atoms [69,79].

### 2.2. Radical-Scavenging Assays

In order to elaborate on the possible reasons for the differing results when comparing scavenging of “non-enzymatic” superoxide KO_2_ to that of “enzymatic” X/XO, a brief review of previously published results [54,55] is provided. The radical-scavenging activity of a compound is illustrated by the CL-SI calculated after the LDCL experiments. It demonstrates the impact of the compounds on luminol-dependent luminescence. The lower the CL-SI, the higher the radical-scavenging activity. The activity in the UA formation was monitored by the relative change in the absorption at 293 nm for a fixed period of time and is presented as a percentage of the same in the absence of the investigated compound. The lower the percentage, the lower the activity of UA formation.

**Figure 2:**
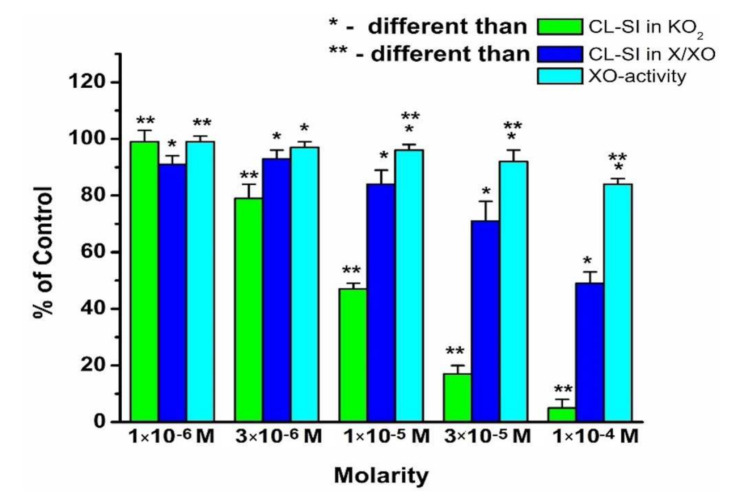
Radical-scavenging activity of HAOA toward superoxide radical formed in KO_2_ and X/XO model systems, and activity of XO in the formation of uric acid (UA) in the X/XO model system. Data = mean ± StDev, *p* < 0.05.

The radical-scavenging activities of HAOA toward superoxide and the effect on the activity of XO to produce uric acid are summarized in Figure 2. All parameters measured were concentration dependent. In the presence of superoxide formed in the KO_2_ model system, HAOA was a pronounced scavenger. Noticeable scavenging activity was observed at concentrations above 1 × 10^−6^ M.

In general, the scavenging activity of HAOA toward X/XO-generated superoxide was weaker than that toward KO_2_ generated superoxide. The enzymatic activity of XO (in terms of UA formation) in the model system X/XO was concentration dependent in a similar way to the scavenging activity. A noticeable difference between O_2_^−^-scavenging activity and activity of UA formation was observed at concentrations of HAOA of 1 × 10^−5^ M and above.

**Figure 3:**
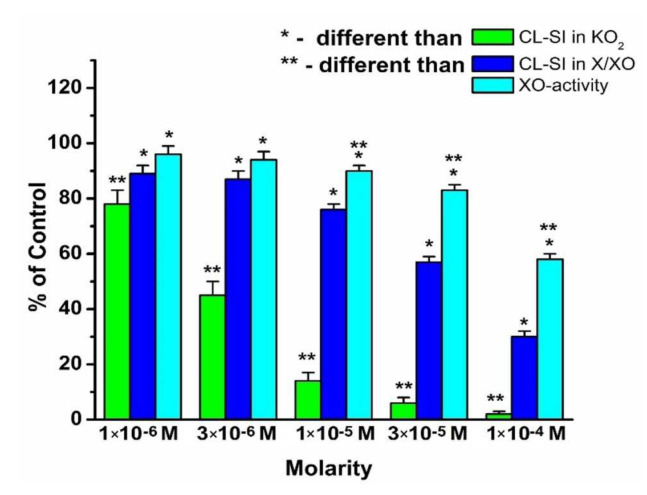
Radical-scavenging activity of GaAOA toward superoxide radicals formed in KO_2_ and X/XO model systems, and activity of XO in the formation of uric acid (UA) in the X/XO model system. Data = mean ± StDev, *p* < 0.05.

The radical-scavenging activity of GaAOA in the presence of the KO_2_ and X/XO model systems as well as the activity of XO in the UA formation are presented in Figure 3. Similar to Figure 2, all parameters observed were concentration dependent. In the presence of the KO_2_ model system, GaAOA behaves as a strong scavenger of superoxide radicals.

Within the entire interval of GaAOA concentrations, the radical-scavenging activity toward KO_2_-generated superoxide was markedly higher than that toward the X/XO-generated radical. The activity of XO to produce UA in the presence of GaAOA was lower than that in the absence of the compound even at GaAOA concentrations as low as 1 × 10^−6^ M. A statistically significant difference between superoxide-scavenging activity and activity of UA formation in the presence of the X/XO model system was observed for GaAOA solutions of concentrations of 1 × 10^−5^ M and above. Figure 2 and Figure 3 demonstrate that in the presence of the X/XO model system, the superoxide-scavenging activity of GaAOA was higher, while UA formation was lower than the same parameters in the presence of HAOA at the same concentration.

**Figure 4:**
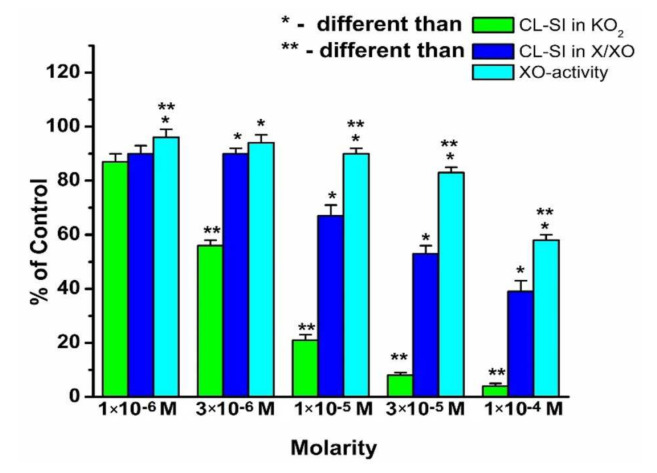
Radical-scavenging activity of LaAOA toward superoxide radicals formed in KO_2_ and X/XO model systems, and activity of XO in the formation of uric acid (UA) in the X/XO model system. Data = mean ± StDev, *p* < 0.05.

The superoxide-scavenging activity and activity of UA formation in the presence of LaAOA are displayed in Figure 4.

All parameters monitored were concentration dependent. Within the entire concentration interval, LaAOA behaves as a scavenger of KO_2_-generated superoxide. At a concentration of 1 × 10^−6^ M, the CL-SI was about 85–90%. In the presence of the X/XO model system, CL-SI decreased with the increase in the concentration of the complex. Above a concentration of 1 × 10^−6^ M, the O_2_^−^-scavenging activity of LaAOA in the X/XO model system was much lower than that in the KO_2_ model system. The activity of UA formation in the X/XO model system decreased with the increase in LaAOA concentrations. In the presence of 1 × 10^−5^ M LaAOA and higher, the activity of UA formation was markedly higher than the CL-SI for X/XO-generated superoxide. Figure 2 and Figure 4 demonstrate that in the presence of the X/XO model system, the radical-scavenging activity toward superoxide was higher, but the activity of the UA formation was lower than the corresponding parameters for same concentration of HAOA.

### 2.3. Molecular Docking Assay

We docked HAOA, GaAOA, and LaAOA into human xanthine oxidase (HXO) with PDB ID: 2CKJ. The docking center was set on Glu1262, Arg913, Gln768, and Arg881 according to Enroth et al. [93,94,95].

**Figure 5:**
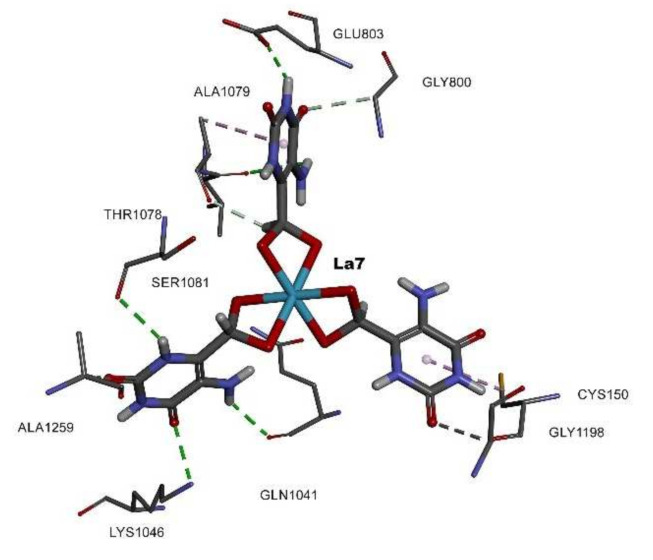
The 3D structure of the lanthanum (III) complex presented as sticks and colored by CPK with the involved amino acids inside the active site of the HXO.

The results showed multiple solutions with up to 100 poses saved per molecule. The best GOLD PLPChem score was selected. The saved PLPChem scores were 34.42, 33.35, and 16.45 for lanthanum(III), gallium(III) complexes, and HAOA, respectively. The docking results showed that the lanthanum(III) complex interacted via a total of 14 hydrogen interactions, as well as five carbon–hydrogen interactions that were saved with the pyrimidine function, thus proving the importance of this function in maintaining stability inside the active site. Only five hydrophobic interactions were found with Ala and Cys amino acids. Gln768 saved an important distance of 1.30 Å, creating the possibility to form metal coordinations between the inhibitor and the enzyme since the ionic oxygen atoms were responsible for causing interactions with the most catalytic amino acids, thus promoting possible irreversible inhibition (Figure 5).

**Figure 6:**
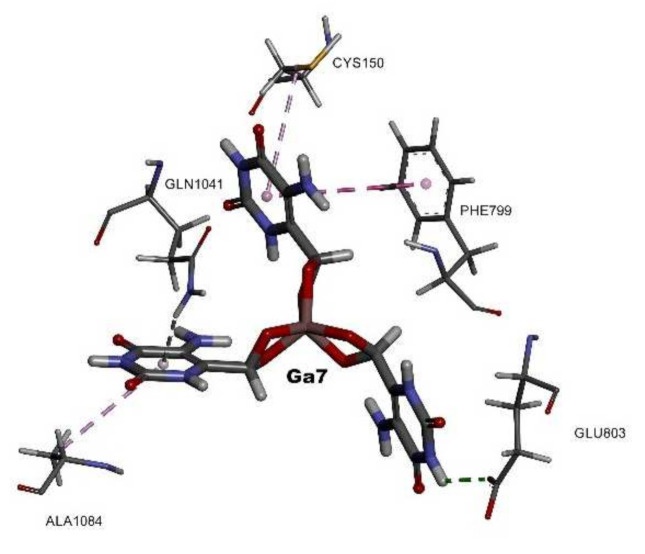
The 3D structure of the gallium(III) complex presented as sticks and colored by CPK with the involved amino acids inside the active site of the HXO.

The gallium(III) complex interacted with eight hydrogen interactions, Gln1041 saved one interaction, pi-donor hydrogen bond type, with the pyrimidine function and three hydrophobic interactions with Ala, Cys, and Phe. The mechanism of action of the gallium(III) complex saved was the same as for the lanthanum(III) complex since the only difference was the ion type, however, the chosen conformation to bind in the active site was slightly different with a repeated ratio (RR) of 90% (Table 1), which could affect the overall inhibition process (Figure 6).

HAOA (Table 3) forms one hydrogen interaction, the conventional hydrogen bond type, saved by Arg913 and one pi–sulfur interaction saved by Met1039 with the uracil group. The major difference between the two complexes (La and Ga) and HAOA was the number of uracil groups (three in the complexes), which can favor more interactions with the active site amino acids.

We docked allopurinol in the HXO in our previous work [96,97] as the reference inhibitor to compare it with the complexes under investigation.

## 3. Discussion and Conclusions

In summary, Figure 2, Figure 3 and Figure 4 show that all three compounds behave as scavengers of superoxide radicals, generated in the presence of the KO_2_ and X/XO model systems in a concentration-dependent manner. The relative differences between the superoxide-scavenging activities of these compounds were in agreement with their previously observed affinity to participate in interactions with stable free radicals via electron transfer reactions [54,55].

The scavenging activity toward X/XO-generated superoxide of each compound at the same concentration is lower than that toward the KO_2_-generated superoxide. It was proposed that this observation might be related to some kind of impact of the compounds on the activity of the enzyme to produce uric acid, as the O_2_^−^ radical is a by-product of UA formation. To explore this probability, a model system was created by using a molecular docking protocol that allows the molecules of HAOA, GaAOA, and LaAOA to interact with the molecule of xanthine oxidase. The reasoning behind this approach lies in the fact that the structure of 5-aminoorotic acid includes a pyrimidine ring that, theoretically, would allow the compound to adsorb onto the enzyme.

The standard inhibitor (allopurinol) exhibited interactions with the catalytic amino acids inside the active site via three hydrogen bonds, whereas the studied inhibitors have one to fourteen hydrogen bonds. The best inhibitor was chosen based on the highest PLPChem score, firstly, and secondly by the ratio of the most repeated poses (RR) as a percentage compared to the control (allopurinol), so, based on these results, LaAOA was the best inhibitor model for XO and GaAOA was moderate, while HAOA was the weakest inhibitor. Based on the present study, the following conclusions can be drawn:

1. HAOA, LaAOA, and GaAOA are all scavengers of superoxide, derived from KO_2_ and X/XO;
2. All three substances manifest higher superoxide-diminishing activity in the non-enzymatic model, compared to the enzymatic model;
3. All three substances diminish the production of UA in the X/XO model. Previously, we proposed that this may be due to an interaction between the substances and one/both elements of the model system;
4. Molecular docking suggested that all three substances interact with the active sites of XO, showing potential as enzyme inhibitors. The calculated strength of the interaction decreases in the order LaAOA > GaAOA > HAOA. This result corresponds well with the data from the XO activity UV assay—HAOA decreases UA formation by 15–20%, while both complexes cause a decrease of about 40–45%.
5. In the enzymatic models, superoxide diminishment is greater than the suppression of UA production. Since, for one molecule UA produced, one superoxide ion is generated, we can propose with a reasonable amount of certainty that in the X/XO model, HAOA, GaAOA, and LaAOA act as both:
  - superoxide scavengers, which is clear enough from the results, derived from the KO_2_ assay;
  - XO inhibitors, a proposition supported by the decrease in UA production in the X/XO model and also by the molecular docking assay. Another possible confirmation of that hypothesis comes from the fact that as molecular docking identified HAOA as the substance with weakest interaction with XO, that same substance caused the least diminishing of UA production in the enzymatic model.

## 4. Materials and Methods

### 4.1. Synthesis of the Complexes

The compounds used for the synthesis of the complexes were Merck products, p.a. grade: La(NO_3_)_3_·6H_2_O and Ga(NO_3_)_3_·6H_2_O. 5-Aminoorotic acid (Figure 1) was used as a ligand. The complexes were obtained via a reaction of lanthanum(III) and gallium(III) inorganic salts and the ligand, in amounts equal to a metal:ligand molar ratio of 1:3. The synthesis was carried out by adding aqueous solutions of La(III) and Ga(III) nitrates to the aqueous solution of the ligand, subsequently raising the pH of the mixture gradually to ca. 5.0 by adding dilute solution of NaOH. The formation of the metal complexes can be represented as the following chemical equations (1), (2):

(1) $$\mathrm{HAOA}⇄H^{+}+{\mathrm{AOA}}^{-}$$

(2) $${\left[M{\left(H_{2}O\right)}_{n}\right]}^{3+}+3{\mathrm{AOA}}^{-}\to \;M{\left({\mathrm{AOA}}^{-}\right)}_{3}\cdot 3H_{2}O$$

where M = La, Ga; HAOA = C_5_N_3_O_4_H_5_; and AOA^−^ = C_5_N_3_O_4_H_4_^−^.

The reaction mixture was stirred with an electromagnetic stirrer at room temperature for one hour. As a result of mixing of the solutions, the precipitates of the obtained complexes were derived. They were filtered (pH 5.0), washed with water, and dried in a desiccator to a constant weight. The obtained complexes were very slightly soluble in water, methanol, and ethanol and highly soluble in dimethyl sulfoxide (DMSO).

### 4.2. Analytical and Spectroscopic Methods

The carbon, hydrogen, and nitrogen contents of the obtained complexes were determined by elemental analysis. Solid-state infrared spectra of the studied compounds were recorded in KBr in the 4000–400 cm^−1^ frequency range by an FTIR IFS25 Bruker spectrometer. Raman spectra of 5-aminoorotic acid and its new La(III) and Ga(III) complexes were recorded with a Dilor microspectrometer (Horiba-Jobin-Yvon, model LabRam) equipped with 1800 grooves/mm holographic grating. The 514.5 nm line of an argon ion laser (Spectra Physics, model 2016) was used for the probes’ excitation. The spectra were collected in backscattering geometry with a confocal Raman microscope equipped with an Olympus LMPlanFL 50× objective and a resolution of 2 cm^−1^. The detection of Raman signal was carried out with a Peltier-cooled CCD camera. Laser power of 100 mW was used in our measurements. ^1^H NMR spectra were recorded at room temperature on a Brucker 250 WM (250 MHz) spectrometer in DMSO-*d_6_*. Chemical shifts are given in ppm, downfield from TMS.

### 4.3. Radical-Scavenging Assays

All materials and compounds were of the finest grade (p.a.) (Sigma-Aldrich). Solutions were prepared in bi-distilled water. The tested concentrations of 5-aminoorotic acid and its complexes with Ga(III) and La(III) were between 1 × 10^−6^ M and 1 × 10^−4^ M. The desired concentrations were achieved by dilution of standard aqueous solutions with concentrations 1 × 10^−3^ M for HAOA and LaAOA and 3 × 10^−4^ M for GaAOA (the highest concentration possible for GaAOA in water). Then, 25.4 mU/mL xanthine oxidase was dissolved in 50 mM K,Na-phosphate buffer with Ph = 7.45 (PBS) and used in the LDCL and UV–Vis measurements. Directly prior to use, 1 mM KO_2_ solution in dehydrated DMSO was prepared. A 3 mM solution of xanthine was prepared by dissolving the compound in 0.1 N NaOH and further dilution with bi-distilled water. 5-Amino-2,3-dihydro-1,4-phthalazinedione (luminol) was dissolved in a small amount of 0.01 M NaOH, further diluted to 1 × 10^−3^ M in 50 mM PBS of pH = 7.45, and pH was adjusted again to 7.45.

LDCL was applied in order to estimate the radical-scavenging activity in the presence of the model systems containing KO_2_ and X/XO. LUMAT LB9507 apparatus was used for the LDCL investigations. The kinetics were measured with delay time 2 s, measuring time 3 s for a total period of 600 s. The integral intensities for the first 10 s were used in data management.

The specific activity of XO in the model system X/XO was estimated by UV spectrophotometric measurements of the relative change in the characteristic signal of uric acid (UA) at 293 nm. This experiment was performed using a UV 1650PC Shimadzu spectrophotometer. The delay time was 10 s, and the activity of the UA formation was computed by the program subroutine for a period of 10–90 s.

#### 4.3.1. Assay for CL in the Presence of KO_2_

One milliliter of the control volume contained 0.05 mL KO_2_ solution, 0.05 mL luminol, and PBS. One milliliter of the sample volume contained 0.05 mL KO_2_, 0.05 mL luminol, the compound investigated in the desirable concentration, and PBS. The results were presented as the chemiluminometric scavenging index (CL-SI), calculated as follows (Equation (3)):

(3) $$\mathrm{CL-SI}\;=\frac{I_{sample}}{I_{control}}\;\times \;100$$

where *I_control_* and *I_sample_* are the integral intensities measured for the KO_2_ alone and in the presence of the compound in a desired concentration. The background measurement showed an integral intensity of 10 and was subtracted from both control and sample measurements. For each compound at each desirable concentration, 5 parallel measurements were performed. Average values and standard deviations were used for further comparisons.

#### 4.3.2. Assay for CL in the Presence of X/XO Model System

The 1 mL cuvette for the control measurement contained 0.02 mL XO solution, 0.1 mL xanthine, 0.1 mL luminol, and PBS. A 1 mL solution for the sample measurement contained 0.02 mL XO, 0.1 mL X, the compound investigated in the desired concentration, and PBS. The CL-SI was determined using the same formula as shown for the KO_2_ assay. For each desirable concentration of the compounds investigated, 5 parallel measurements were performed. The average values and standard deviations were calculated and used in further comparisons.

#### 4.3.3. Assay for UV Determination of XO Activity in the Presence of the X/XO Model System

One milliliter of the reaction mixture for the control measurement contained 0.02 mL XO, 0.1 mL xanthine, and PBS. The 1 mL cuvette for the sample measurement contained 0.02 mL XO, 0.1 mL X, the compound investigated in a desired concentration, and PBS. As no additional components in this model system were present, only UA and O_2_^−^ were produced. As the final product of xanthine transformation was uric acid (UA), the absorption at 293 nm (characteristic wavelength for UA) was measured for 10 min, using the molar extinction coefficient of 1.22 × 10^4^ M^−1^·cm^−1^ [98]. The activity of XO was defined as the amount of enzyme needed to convert 1 µmole of xanthine for 1 min in a 1 mL reaction mixture at 298 K. Data for the sample activity of XO were presented as a percentage of the XO activity seen in the control measurement.

### 4.4. Statistical Analysis

Five parallel measurements were performed for each investigated concentration of every substance. Each measurement represented an individual data point. Averages and standard deviations were calculated. Relative changes within the limits of experimental error were ignored. The significance of differences between standard deviations was verified by the Bartlett test. One-way ANOVA, followed by the Bonferoni post-test confirmed the concentration-dependent impact of the substances on the radical-generating model systems. The Bartlett test was used to verify that all standard deviations belonged to the same population. Differences at *p* < 0.05 were considered as statistically significant.

#### In Silico Molecular Docking Assay

The molecular docking protocol was chosen to be a specific docking type; in this case, full flexibility was given to the inhibitor. The Genetic Optimization Ligand for Docking software (GOLD) [99] protocol involved six steps:

- removing ligands, heteroatoms, and unnecessary water molecules unless needed at the active site (step I);
- checking the receptor ionization and tautomeric states by adding the necessary hydrogen atoms (step II);
- defining the active site by using the list of active site residues mode (step III);
- setting the ligands by performing energy minimization using the Hyperchem v8.0 program [100]. First, we used the single point mode to calculate the ligand energy and gradient, then molecular mechanics optimization was performed using the Polack–Ribiere conjugate gradient algorithm where the RMS gradient of 0.1 kcal/(Å mol) was used for 735 cycles (step IV);
- fitness function choice, and the piecewise linear potential (PLPChem score) was chosen as the most recommended fitness function in GOLD [99] (step V);
- the number of genetic algorithm (GA) runs, and 100 solutions (results) per inhibitor were chosen as the most accurate (step VI).

The root mean square deviation (RMSD) and cluster size were kept as the default settings [101]. The best solutions were chosen based on the highest PLPChem score, along with the ratio of the most repeated poses as a percentage. The obtained molecular docking results were analyzed using Discovery Studio Visualizer (DSV), v 4.0 [102].

PLP and ChemPLP are empirical fitness functions, optimized for pose prediction; they model the attraction as well as repulsion of protein and ligand heavy atoms. In both cases, the piecewise linear potential (PLP) is utilized to model the steric complementarity between protein and ligand, while ChemPLP adds the distance- and angle-dependent hydrogen and metal bonding terms. The internal score of the ligand consists of the heavy-atom clash potential (*flig-clash*) [99,103,104] as well as the torsional potential used within ChemScore (*flig-tors*). Both fitness functions are capable of covalent docking (*fchem-cov*), considering flexible side chains (*fchem-prot*) and explicit water molecules as well as handling constraints (*fcons*) [105]. The Discovery Studio Visualizer program was used for result treatments.

## Figures and Tables

**Table 1:** Selected experimental IR and Raman wavenumbers (cm^−1^) of 5-aminoorotic acid (HAOA) and its La(III) and Ga(III) complexes [LaAOA, GaAOA] and their tentative assignment. Abbreviations: vw—very weak; w—weak; m—medium; ms—medium strong; s—strong; vs—very strong; sh—shoulder; ν—stretching; δ—bending; τ—torsion; s—symmetric; as—asymmetric; def.—deformation; ip—in plane; op—out of plane; ring—pyrimidine ring; sciss—scissoring; wagg—wagging; M—metal.

IR			Raman			Vibrational Assignment
**HAOA**	**LaAOA**	**GaAOA**	**HAOA**	**LaAOA**	**GaAOA**	
3457 s	3479 w	3443 w	3479 w	3478 vw		ν(N1H1)
	3361 m	3355 m	3456 vw	3357 w	3358 w	ν_as_(NH_2_)
3333 s	3448 w	3336 m	3323 m		3334 w	ν(N3H3), ν(C-H)
3196 m	3171 m	3168 m	3166 vw			ν_s_(NH_2_)
1691 vs	1718 m	1717 m	1698 m			δ(NH); ν_s_(C2=O2), ν(N–C6)
	1684 vs	1691 vs				ν_s_(C4=O4)
1667 s	1673 vs	1676 vs	1678 sh	1673 m	1682 m	ν_s_(C4=O4); ν(COO^−^), ν(C5=C6), δ(N3–H3)
1604 s	1637 vs	1645 vs	1612 vs	1623 vs	1628 vs	ν(C5=C6); β(NH_2_), ν(COO^−^)
1566 m	1556 m	1553 m	1560 m	1542 w/m	1546 w/m	δ_ip_(N1H1, N5H5); ν(C5C6), β_s_(NH_2_)
1511 w	1499 m	1498 m	1492 w/m	1494 w	1501 w	δ(NC); ν(ring); δ_ip_(N3H3)
1457 m			1447 w		1433 w	ν(ring), β_s_(NH_2_), δ(Nl–H1), ν(COO^−^)
1436 m	1424 s	1425 s	1421 w	1420 vs	1407 vs	δ(N3H3), δ(ring), δ(N1H1)
1405 m	1390 s	1391 s		1384 s	1388 s	δ(N3H3), δ(ring), δ(N1H1), ν_s_(COO^−^)
1312 m	1306 m	1306 m	1301 w	1295 m/s	1302 m/s	ν(C5–N), ν(C–N), δ(OH), β_s_(NH_2_)
1255 m/s	1290 sh	1284 sh				ν(C–N), δ(N1H1), r(NH_2_), δ(ring)
1234 m/s	1237 w	1239 w	1242 m	1230 m/s	1240 m/s	ν(C–N), δ(N3H3), r(NH_2_), δ(NlH1)
1140 sh	1122 vw	1126 vw		1124 vw	1131 vw	δ(OH)
1083 vw			1047 vw	1041 w	1044 w	ν(C6-O, C6-C7), β_as_(NH_2_)
989 sh						ν(NCN), δ(N3H3), r(NH_2_), δ(NlH1)
924 w	941 sh	944 sh	919 w/m	933 w/m	948 w/m	ν(NCC), ν(ring), r(NH_2_), ν(COO)
871 w/m	884 vw	875 vw				γ(N3–H3), γ(ring)
795 vw	798 m/807	797 m/807	809 vw	788 sh	781 sh	δ_op_(O3C7O1)
767 vw	775 w	777 w		763 m	777 m	γ(C4=O4), γ(C4–C=C6), γ(C6–C12)
754 w	759 w	759 w	749 vw			γ(C6–C12), γ(C4=O4), γ(COOH), γ(N3–H)
740 w	747 w	749 w				γ(C2=O2), γ(NC2N), γ(N3–H)
696 vw	694 vw	692 vw				δ(ring), Δ_s_COO), r(NH_2_)
	617 vw	602 vw				ν(M-O)
584 vw		595 vw	582 w	581 w/m	589 w/m	δ(ring), Δ_s_(COO)
	521 sh	509 sh				ν(M-O)
488 w		501 w	482 w/m	480 m	495 m	δ(ring), δ(NH_2_), Δ_s_(COO)
474 w/m	459 sh	465 sh				γ(OH)
446 m	441 m	442 m	445 w			δ(OCNCO), δ(COO) + r(NH_2_)
	422 sh	422 sh	425 sh	425 w/m	438 w/m	τ(C2O2, ring), Δ_as_(COO)
			381 vw	375 w	374 w	δ(OCCN11), δ(COO), δ(C2=O), r(NH_2_); ν(M-O)
			249 w	339 sh	243 w	τ(NH_2_), Δ_s_(COO)
				224 w	205 w	ν(O-M-O)
			196 w	192 sh	182 sh	τ(ring); δ(O-M-O)

**Table 2:** ^1^H NMR (250 MHz, DMSO-*d_6_*).

N_n_-H	HAOA	LaAOA	GaAOA
N_1_-H	11.47	11.22	11.46
N_3_-H	9.44	9.03	8.48
C_5_NH_2_-2H	6.00	5.57	5.33

**Table 3:** The molecular docking results analysis.

Rank	Inhibitors	RR (%)	PLPChem Score	Nucleophilic Residues	Interaction Type	Length (Å)	Number of Interations	Fav/Unfav Bond
Control	Allopurinol	100	23.89	GLY798	Hydrogen Bond	2.15	04	8/0
					Hydrophobic Bond	4.07	04	
1	LaAOA	100	34.42	GLN768	Hydrogen Bond	1.30	14	20/4
					Hydrophobic Bond	4.24	05	
					Coulombic	3.47	*01*	
2	GaAOA	90	33.35	GLN1195	Hydrogen Bond	1.90	12	15/01
					Hydrophobic Bond	4.46	03	
					Coulombic	/	00	
3	HAOA	100	16.45	ARG913	Hydrogen Bond	3.15	01	3/0
					Hydrophobic Bond	4.38	02	
					Coulombic	/	00	

PLPChem: piecewise linear potential; Fav/Unfav: favorable/unfavorable; RR: repetition ratio.

## Data Availability

Not Applicable.

## Sample Availability

Samples of the compounds are not available from the authors.
